# A decade of neonatal sepsis caused by gram-negative bacilli—a retrospective matched cohort study

**DOI:** 10.1007/s10096-021-04211-8

**Published:** 2021-03-24

**Authors:** Viveka Nordberg, Aina Iversen, Annika Tidell, Karolina Ininbergs, Christian G. Giske, Lars Navér

**Affiliations:** 1 Department of Neonatology, Karolinska University Hospital, Stockholm, Sweden; 2 Department of Clinical Science, Intervention and Technology (CLINTEC), Division of Paediatrics, Karolinska Institutet, Stockholm, Sweden; 3 Department of Clinical Microbiology, Karolinska University Hospital, Stockholm, Sweden; 4 Department of Laboratory Medicine, Division of Clinical Microbiology, Karolinska Institutet, Stockholm, Sweden; 5 Department of Neonatology, Sachs’ Children’s Youth Hospital, Södersjukhuset, Stockholm, Sweden

**Keywords:** Gram-negative bacilli, Sepsis, Neonatal, Antibiotic resistance, Mortality

## Abstract

This study is to determine the incidence and outcome of neonatal gram-negative bacilli (GNB) sepsis in Stockholm, Sweden, and describe bacterial characteristics. This is a retrospective cohort study. All infants with GNB-sepsis between 2006 and 2016 were included and matched with two control groups, with suspected sepsis and uninfected neonates, respectively. Outcome was death before discharge, risk of death within 5 days after sepsis onset, and morbidity. The resistance pattern from all GNB was collected, and all available isolates were subjected to genome typing. All neonates with GNB-sepsis (*n* = 107) were included, and the cumulative GNB-sepsis incidence was 0.35/1000 live born. The in-hospital mortality was 30/107 (28%). GNB late-onset sepsis (LOS) was associated with an increase in mortality before discharge compared to uninfected controls (OR = 3.9; CI 1.6–9.4) but not versus suspected sepsis. The suspected LOS cases did not statistically differ significantly from uninfected controls. The case fatality rate (CFR) at 5 days was 5/33 (15%) in GNB early-onset sepsis (EOS) and 25/74 (34%) in GNB-LOS. The adjusted hazard for 5 days CFR was higher in GNB-LOS versus uninfected controls (HR = 3.7; CI 1.2–11.2), but no significant difference was seen in GNB-LOS versus suspected sepsis or in suspected sepsis versus controls. ESBL production was seen in 7/107 (6.5%) of the GNB isolates. GNB-LOS was associated with a higher 5 days CFR and in-hospital mortality compared to uninfected controls but not versus suspect sepsis. The incidence of both GNB-EOS and GNB-LOS was lower than previously reported from comparable high-income settings. The occurrence of antibiotic resistance was low.

**Supplementary Information:**

The online version contains supplementary material available at 10.1007/s10096-021-04211-8.

## Introduction

Neonatal infections account for more than one-third (36%) of all neonatal deaths globally. Sepsis is the leading cause of neonatal mortality and accounts for more than one million deaths/year worldwide [1, 2]. In high-income settings, the incidence of neonatal sepsis is reported to be 1–4/1000 live births [1, 3]. Among very low birth weight (VLBW) neonates, approximately 30–40% suffer from late-onset sepsis (LOS) with a mortality rate between 10 and 36% depending on the infecting organism [4–6]. Infants with gram-negative bacilli (GNB)-LOS are associated with a higher mortality compared to gram-positive bacteria (GPB)-LOS [7, 8]. Studies from Sweden in the last decade report an incidence of early-onset sepsis (EOS) of 0.9/1000 live births with a case fatality rate (CFR) of 7% and the GNB-EOS incidence of 0.25/1000 live born with a CFR of 13% [9]. There are no previous studies on the incidence or the CFR of neonatal GNB-LOS in Sweden.

The growing challenge of antimicrobial resistance (AMR) in neonatal intensive care units (NICUs), especially with resistant GNB, is associated with a high mortality and poor long-term outcome [7, 10–12]. The spread of antibiotic resistant bacteria has been a persisting clinical problem during the last decades and has resulted in approximately 214,000 attributable neonatal deaths/year globally [13]. A reduction in inappropriate use of antibiotics would be the most important step to decrease AMR. The challenge is to reduce the use of antibiotics without an increase in fatal outcome [14, 15].

Early diagnosis and treatment of neonatal sepsis are difficult, and the fact that a consensus definition of neonatal sepsis is lacking makes it even more challenging [16–18]. The neonatal immune defense, clinical symptoms, and pathophysiologic responses to bacterial infection differ in term and preterm neonates due to age-dependent maturity. Sepsis onset is most rapid in preterm neonates [19–21]. The characteristics of the infecting bacteria, such as virulence and resistance factors, play a role in the dynamics of the infection. A positive blood culture is the gold standard definition of sepsis. However, the difficulties in getting adequate blood volumes for culture and biomarkers with low sensitivity and specificity complicate the sepsis diagnosis [22]. The intestinal dysbiosis, following antibiotic treatment, is associated with a higher risk of LOS, necrotizing enterocolitis (NEC) and other long-term morbidities [23–28].

We aimed to analyze the incidence of neonatal GNB-sepsis and associated mortality and morbidity in neonates in our setting. We also wanted to determine whether there were differences in outcome between patients with culture proven sepsis, suspected sepsis (negative blood culture) and uninfected patients. We characterized the invasive bacterial isolates as to clonality and presence of AMR genes.

## Materials and methods

### Study population and setting

There are six delivery units and four NICUs in the Stockholm region. A total of 29,553 infants were born alive at these delivery units during 2016. The NICUs are Karolinska Danderyd (level 2), Karolinska Solna (level 3), Karolinska Huddinge (level 3), and Södersjukhuset (level 2). From March 2014 to May 2016, a seventh small delivery unit and levels 1–2 neonatal unit, BB Sophia, operated.

The all-cause neonatal mortality before 28 days of life in the Stockholm region was 0.7–1.8/1000 live born (mean 1.4/1000) (2006–2016). The recommended empiric antibiotic therapy for unknown EOS was since 2012 benzylpenicillin/amikacin and for LOS cloxacillin/amikacin or cefotaxime/amikacin [29]. Between 2006-2012 the empiric aminoglycoside was gentamicin or netilmicin, which during 2012 was changed to amikacin due to local outbreaks with gentamicin resistant *E.coli*. Infection control routines were similar in all included hospitals.

### Patients and study design

A matched cohort study was undertaken where all neonates with GNB-sepsis at Stockholm’s four NICUs between January 2006 and December 2016 were included. We identified all patients with a positive GNB blood culture, at least two clinical signs (fatigue, respiratory instability, temperature instability, poor feeding, vomiting, cyanosis) and antibiotic therapy for > 5 days. Patients with GPB-sepsis were not analyzed. EOS and LOS were defined according to age at onset of sepsis symptoms before or after 72 h of age.

### Identifying the cases and the controls

To identify the sepsis cases, we used the ICD-10 codes for GNB-sepsis in the electronic medical record systems Take Care and Clinisoft and merged them with the Swedish Neonatal Quality Register (SNQ). Patient characteristics from the total NICU-stay were collected.

The two groups of controls, manually collected from the same registries, were neonates with suspected sepsis and those uninfected during their NICU-stay. We chose controls with the same gestational age (GA) and closest birth date. Suspected sepsis was defined as the ICD-10 code P36.9, clinical symptoms, a negative blood culture, and subsequent antibiotic therapy for at least 5 days. Uninfected infants, alive at 72 h of age, were defined as not fulfilling the ICD-10 criteria for sepsis or suspect sepsis during the NICU-stay. The proportion of the case vs suspected sepsis vs uninfected was planned to be 1:1:3. The number of uninfected neonates in the same gestational ages as the cases during 2006–2016 was insufficient; hence, the proportion of the cases to controls was 1:1:2.6.

There was no variability of GA in the matching groups, but there was variability in closest birth date in the controls depending on GA. The variability in closest birth date between cases in the three matching groups was 10 years, but on average below 24 months.

### Outcomes and definition of sepsis-related mortality

The primary outcome was death before discharge from NICU. The secondary outcomes were sepsis mortality 5 days after onset of GNB-LOS and major morbidities, such as retinopathy of prematurity (ROP), intraventricular hemorrhage (IVH), and bronchopulmonary dysplasia (BPD).

Death 5 days following a positive blood culture is presented in the study as 5 days case fatality rate (CFR). The suspected sepsis CFR was death 5 days after onset of therapy for suspected sepsis. Repeated episodes of sepsis were documented in a few numbers of patients, but survival was analyzed from the first invasive GNB episode. Proportions of multidrug-resistant GNB strains and the burden of AMR in clinical samples were determined.

### Selection of bacterial isolates

GNB in the study refers to the following species: *Escherichia coli*, *Klebsiella pneumoniae*, *K. oxytoca*, *K. aerogenes*, *Enterobacter cloacae*, *Citrobacter koseri*, *Serratia marcescens*, *Proteus mirabilis*, *Pseudomonas aeruginosa*, *Acinetobacter baumannii*, and *Haemophilus influenzae*. The *Neisseria* species were considered to be contaminants. Blood cultures with contaminants were not included. All isolates were susceptibility tested for the following: gentamicin, amikacin, trimethoprim-sulfamethoxazole, cefotaxime, ceftazidime, ciprofloxacin, imipenem, meropenem, ertapenem, and piperacillin-tazobactam.

### Characterization of gram-negative bacilli

All GNB isolates were cultured, isolated, and identified according to routine validated clinical methods and guidelines used during the study period. Antibiotic susceptibility testing was performed by the disk diffusion method and interpreted according to the guidelines of the Swedish Reference Group of Antibiotics before 2011 and between 2011 and 2016 according to guidelines from the European Committee on Antimicrobial Susceptibility Testing (www.eucast.org). MDR was defined as resistance to at least one antibiotic agent in three or more antibiotic groups [30].

Due to the retrospective design of the study, only 33/107 isolates were available for the genetic analyzes. Whole genome sequencing (WGS) was performed at the Science for Life Laboratory (SciLife, Solna, Sweden). Multi-locus sequencing (MLST) was performed in silico as described previously [31]. All *Enterobacterales* were assigned to sequence types except *S. marcescens*. The isolates that were closely related in the MLST analysis were further analyzed with single nucleotide polymorphism (SNP) analysis in CLC Workbench [31].

### Statistical methods

This was an open cohort design with varying lengths of time from onset of sepsis to discharge. Comparisons of continuous variables were made with Wilcoxon rank-sum or two sample *t*-test and summarized using means and SDs if unimodal, symmetrically distributed variables. If the distribution was skewed, they were shown with median values and ranges. Pearson’s Chi-squared test was used to compare categorical variables. Statistical significance was defined as *p* values < 0.05, and confidence intervals of 95% were used.

We used logistic regression to measure odds ratios (OR) of dying, separately for EOS and LOS, and adjusted for different variables in the regression model of EOS and LOS. Variables adjusted for in the EOS group were as follows: gestational age, gender, perinatal antibiotics, birth mode, and prenatal steroid treatment. In the LOS group, we adjusted for gestational age, gender, prenatal steroid treatment, mechanical ventilation, and necrotizing enterocolitis (NEC). We chose these variables since mechanical ventilation is associated with a higher mortality and prenatal steroids with a lower mortality generally. We also adjusted for NEC since we considered NEC to be a confounder in the association between GNB-sepsis and death. Because of the strong correlation between birth weight (BW) and GA, the risk factor BW was excluded from the analysis. In the logistic regression for morbidities (ROP, IVH, BPD), we used composite binary variables for death and the specific morbidity.

We analyzed EOS and LOS separately in the survival analysis. The Kaplan-Meier method was used to visualize survival over time. In the survival analysis for 5-day mortality after index day (GNB-sepsis date of the respective case), Cox proportional hazard regression was performed to measure the hazard ratio (HR) for dying between the cases and their matched controls. The HR gives the time-dependent instantaneous rate ratio of dying, but is in this study interpreted as a ratio of risks of death occurring within 5 days, similar to the interpretation of ORs in logistic regression.

Covariates adjusted for in the Cox-regression model in the EOS and LOS cohort were the same as in the logistic regression model. Stata Statistical Software version 16.0, StataCorp, TX, USA, and JMP 15.1.0. SAS Institute Inc., Cary, USA, were used.

## Results

During the study period, 310,091 infants were born alive at the included delivery units. Of these, 31,878 (10.2%) neonates were admitted to the neonatal units of Karolinska Danderyd (*n* = 10,418), Karolinska Solna (*n* = 5828), Karolinska Huddinge (*n* = 6904), and Södersjukhuset (n = 8728). These four units are levels 2–3 NICUs with a total of 75–80in-patient cots.

### Incidence of GNB-sepsis and baseline characteristics

**Fig. 1:**
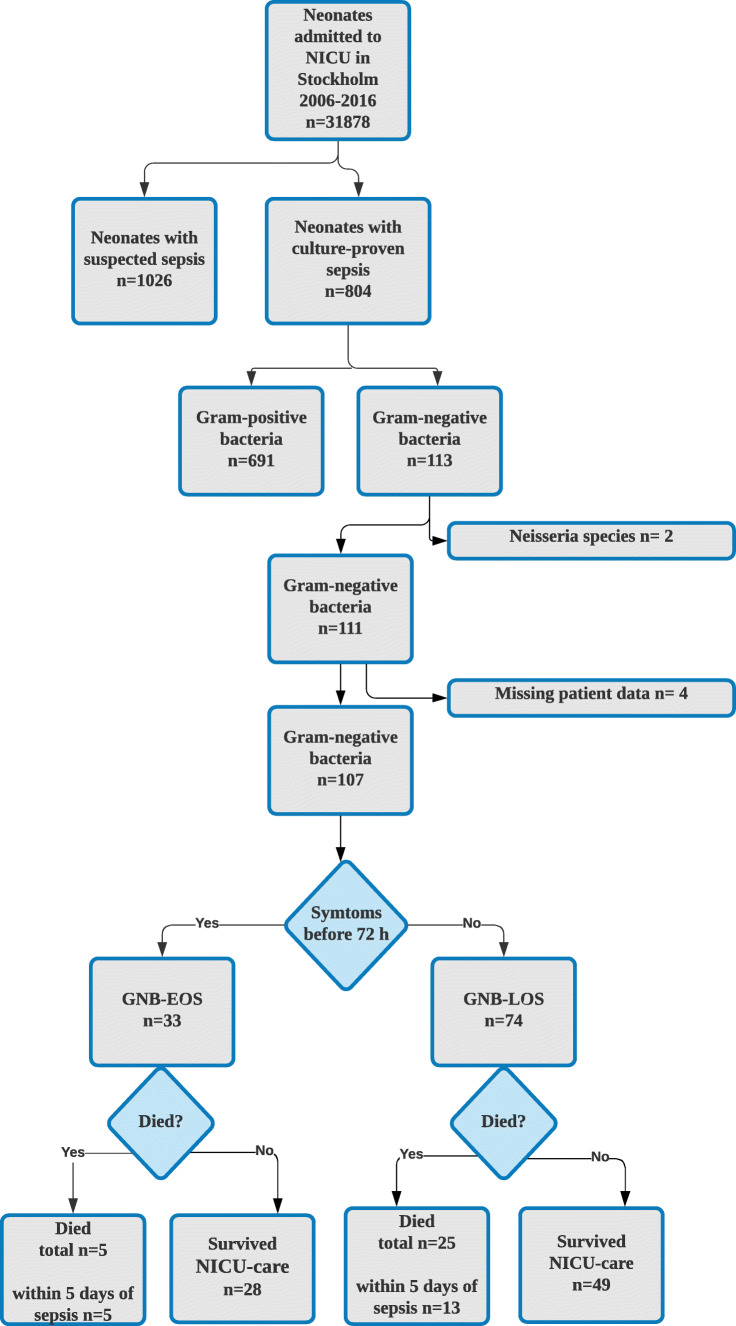
A Flow chart of all included patients in the study

A flowchart of included patients is depicted in Fig. 1 during the period, a total of 804 admitted infants had a culture-confirmed neonatal sepsis, which corresponds to a total incidence of 2.6/1,000 live born. GNB-sepsis counted for 111/804 (14%) of all culture-confirmed sepsis cases.

The proportion of GNB-sepsis for all admitted neonates was 111/31,878 (0.36%), with a cumulative incidence of 0.35 cases per 1000 live born during the study period. Among the infants admitted to the neonatal unit, 1026/31,878 (3.2%) had suspected but not culture-verified sepsis with a cumulative incidence of 3.3/1,000 live born.

Among neonates with invasive GNB-sepsis (*n* = 111), medical records were retrievable in 107 patients, of which 33 were GNB-EOS and 74 were GNB-LOS. These cases were matched with 107 patients with suspected sepsis (culture-negative) and 295 uninfected controls. In total, data from 509 patients were analyzed. The clinical characteristics of included patients are presented in Table 1.

**Table 1:** Characteristics of 107 GNB cases (EOS and LOS) and pair-wise comparisons with suspected sepsis controls and uninfected controls

	GNB-EOS (*n* = 33)	Susp EOS (*n* = 33)	No inf control (*n* = 99)	P* EOS	P** EOS	P*** EOS	GNB-LOS (*n* = 74)	Susp LOS (*n* = 74)	No inf control (*n* = 196)	P* LOS	P** LOS	P*** LOS
Gest age (w)	34 (26–38)	34 (26–38)	34 (26–38)	0.94	0.97	0.91	27 (25–29)	27 (25–29)	27 (25–29)	0.98	0.60	0.58
Gender, male	16 (48)	21 (64)	57 (58)	0.16	0.36	0.42	45 (61)	53 (72)	99 (51)	0.16	0.13	0.002
BW (g)	2225 (994–2950)	2013 (686–3500)	2087 (995–3325)	0.88	0.81	0.89	885 (750–1395)	812 (627–1259)	960 (747–1315)	0.17	0.69	0.04
Apgar at 5 min < 7	13 (39)	5 (15)	26 (26)	0.027	0.16	0.18	23 (31)	29 (39)	63 (32)	0.33	0.83	0.33
Caesarean section	16 (48)	16 (48)	50 (22)	0.90	0.96	0.84	31 (42)	11 (15)	44 (22)	< 0.001	< 0.001	0.17
Prenatal steroids	11 (33)	14 (42)	34 (34)	0.72	0.72	0.40	52 (70)	64 (87)	146 (75)	0.17	0.61	0.034
Antenatal antibiotics	17 (52)	11 (33)	16 (16)	0.11	< 0.001	0.037	36 (49)	31 (42)	79 (40)	0.048	< 0.01	0.81
Onset sepsis (d)	1 (0–1)	0 (0)		0.023			19 (11–31)	9 (4–17)		< 0.001		
Days of MV	1 (0–7)	0 (0–6)	0 (0)	0.77	< 0.001	0.57	8 (2–24)	9 (2–16)	0 (0–7)	0.43	< 0.001	< 0.001
Days of CPAP	2 (0–8)	1 (0–4)	3 (0–20)	0.31	0.048	0.24	16 (3–36)	18 (2–38)	6 (1–25)	0.77	0.003	0.03
Days of TPN	8 (2–13)	2 (0–10)	1 (0–8)	0.06	0.001	0.001	22 (11–37)	13 (8–24)	9 (5–14)	0.001	< 0.001	< 0.001
Days of UAC	1 (0–6)	0 (0–2)	0 (0–4)	0.038	0.046	NA	6 (3–8)	5 (0–7)	3 (0–6)	0.43	< 0.001	0.036
Days of UVC	1 (0–5)	0 (0–1)	0 (0–0)	0.062	0.015	NA	1 (0–4)	2 (0–5)	0 (0–5)	0.40	0.72	0.20
Days of pCVC	0 (0–9)	0 (0–9)	0 (0–0)	0.39	< 0.001	0.55	15(8–28)	7 (1–20)	2 (0–8)	0.009	< 0.001	< 0.001
BPD discharge	4 (12)	6 (18)	13 (13)	0.64	0.67	0.78	32 (43)	35 (47)	64 (33)	0.48	0.12	0.083
ROP 1–2 discharge	4 (12)	6 (18)	2 (2)	0.48	0.004	0.059	9 (12)	20 (27)	26 (13)	0.11	0.99	0.017
ROP 3–4 discharge	4 (12)	0 (0)	1 (1)	0.095	0.01	0.85	10 (14)	4 (5)	9 (5)	0.18	0.019	0.83
IVH 1–2 discharge	7 (21)	4 (12)	5 (5)	0.32	0.005	0.059	16 (22)	14 (19)	19 (10)	0.55	0.008	0.039
IVH 3–4 discharge	5 (15)	3 (9)	2 (2)	0.48	0.004	0.059	6(8)	6 (8)	11 (6)	0.60	0.20	0.45
All NEC							27 (36)	15 (20)	5 (3)	0.09	0.059	0.39
Surgical NEC							11 (15)	5 (7)	0 (0)	0.11	0.003	0.78
Mortality	5 (15)	3 (9)	7 (7)	0.46	0.24	0.86	25 (34)	12 (16)	15 (7.6)	0.04	< 0.001	0.008

*Comparison between case and suspect sepsis, **comparison between case and uninfected control, ***comparison between suspected case and uninfected control. Continuous variables are presented with means, SD, medians, and interquartile range. Categorical variables are presented with proportions and %. *BW* birth weight, *MV* mechanical ventilation, *CPAP* continuous positive airway pressure, *TPN* total parental nutrition, *pCVC* peripheral central venous catheter, *UVC* umbilical venous catheter, *UAC* umbilical arterial catheter, *BPD* bronchopulmonary dysplasia, *ROP* retinopathy of the newborn, *IVH* intraventricular hemorrhage

More than one LOS episode was seen in 35/107 (33%) cases where the causative pathogens were GNB and GPB, and 57% (20/35) of them had a GPB-sepsis episode before a GNB-sepsis. The 33 GNB-EOS cases were distributed as 4, 4, 1, 3, 2, 4, 4, 5, 1, 2, and 3 per year during the years 2006–2016. There was no statistical difference in the trend of EOS cases per year during the study period. The 74 GNB-LOS cases were distributed as 4, 9, 12, 5, 11, 8, 5, 3, 4, 5, and 8 which indicate a slight but not statistically significant decrease over the period.

The pairwise analysis between groups showed that the median age at diagnosis was 1 day for GNB-EOS, 0 for suspected EOS (*p* = 0.023), 19 days for GNB-LOS, and 9 for suspected LOS (*p* < 0.001).

The administration of prenatal steroids did not differ between culture proven GNB-EOS and suspected sepsis. The GNB-EOS group did not differ from the suspected EOS group regarding administration of antibiotics to mothers prenatally (*p* = 0.11), but the GNB-EOS group had a significantly higher use compared to the uninfected group (52% vs 33 %). Similar results were found in GNB-LOS (49%) where use of antenatal antibiotics differed from their uninfected control group (40%) (*p* < 0.01).’

There were 43/74GNB-LOS cases vs 41/74 suspected LOS cases that received prophylactic antibiotics before the sepsis/suspected sepsis episodes. Mode of delivery did not differ between the groups in the GNB-EOS analysis, but caesarean section was significantly more common in GNB-LOS (42%) compared to suspected LOS (15%) and uninfected controls (22%) (Table 1).

### Intensive care interventions

The median days of mechanical ventilation differed between GNB-EOS cases (median 1 day, IQR 0–7 days) and uninfected cases (median 0, IQR 0–0 days). The days of total parental nutrition (TPN) in the GNB-EOS (median 8 days, IQR 2–13) days were higher and differed significantly from the uninfected group (median 1 day, IQR 0–8 days).

GNB-LOS and suspected LOS had significantly more days of ventilatory support, umbilical artery catheter (UAC), peripheral central venous catheter (pCVC), and TPN than the uninfected group. Days of TPN and total days with pCVC were significantly higher in the GNB-LOS group compared to the suspected sepsis and the uninfected group (Table 1).

### Mortality

Thirty (30/107) neonates with GNB-sepsis died before discharge (5/33 EOS and 25/74 LOS), with a case fatality rate of 28%. The median age at death was 28 days (IQR 14–52) among the infants with GNB-LOS that died during hospital stay. The mortality in the EOS group was too small to make univariate comparisons between the groups relevant. Comparing GNB-LOS with the suspected sepsis and uninfected control groups, the proportion of deaths before discharge was 33.7% (25/74), 18.9% (14/74), and 7.6% (15/196), respectively. The CFR of GNB-LOS in different gestational ages were in GA ≤ 28 (18/52, 35 %), GA 29–32 (6/17, 35%), GA 33–36 (1/3, 33%), and GA ≥ 37 (0/2, 0%). Proportions of deaths of GNB-EOS and GNB-LOS in different gestational ages are presented in Online Resource 1.

In the logistic regression of the relation between GNB-LOS and death, there was a 2.2 times higher odds (crude OR) of dying before discharge at NICU in the GNB-sepsis group (EOS and LOS combined) compared to the suspected sepsis group and 4.8 times higher odds compared to uninfected cases. There was no statistically significant difference in mortality before discharge between patients with GNB-EOS, suspected EOS, and controls. Gestational age was the only factor associated with death in GNB-EOS(Table 2).

**Table 2:** Logistic regression with adjusted odds ratio of neonatal death after GNB-sepsis before discharge from NICU

EOS	Adjusted OR	95% CI	*p* Value	LOS	Adjusted OR	95% CI	*p* Value
GNB-EOS: uninfected*	2.5	0.53–11.4	0.25	GNB-LOS: uninfected*	3.9	1.61–9.36	0.003
Suspected EOS: uninfected*	0.92	0.18–4.74	0.92	Suspected LOS: uninfected*	2.0	0.78–5.05	0.15
GNB-EOS: suspected EOS*	2.7	0.49–14.7	0.26	GNB-LOS: suspected LOS*	2.0	0.82–4.65	0.13
Gestational week	0.8	0.67–0.95	0.01	Gestational week	0.8	0.67–0.92	0.002
Gender (male)	0.9	0.26–2.84	0.81	Gender (male)	1.8	0.87–3.62	0.12
Prenatal steroids	0.7	0.13–3.79	0.67	Mechanical ventilation	3.8	1.00–14.1	0.049
Prenatal antibiotics	2.1	0.50–8.56	0.32	Prenatal steroids	0.4	0.16–0.89	0.26
Birth mode (CS)	1.4	0.37–5.50	0.60	Necrotizing enterocolitis	3.0	1.34–6.48	0.007

Adjusted odds ratio of the comparisons between the sepsis group and the reference groups. GNB-EOS and GNB-LOS are reported separately. *Reference group

Neonates with GNB-LOS were 6.5 (crude OR) and 3.9 (CI: 1.6–9.4) (adjusted OR) more likely to die during hospital stay compared to the uninfected matched control group. A higher gestational age was protective. The comparison between GNB-LOS and suspected LOS showed no significant difference in the odds of dying before discharge (OR 2.0; CI: 0.8–4.6) (Table 2).

**Fig. 2:**
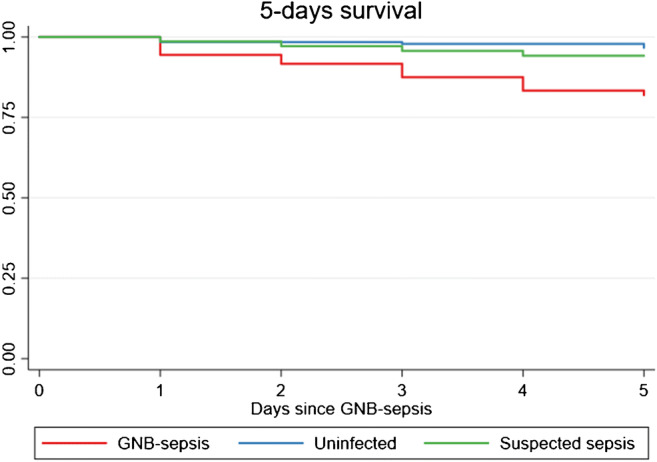
The Kaplan-Meier method visualizes survival over time in GNB-LOS. The figure depicts survival 5 days after sepsis onset

The 5 days CFR was 15% (5/33) in GNB-EOS. All neonates with GNB-EOS that died died before 5 days after GNB-EOS onset. The 5 days CFR of GNB-LOS was 17.6% (13/74). The crude 5 days CFR differed significantly between GNB-LOS and the uninfected controls (*p* < 0.001) and between GNB-LOS and the suspected sepsis group (*p* = 0.039) but not between the suspected sepsis group and uninfected controls (*p* = 0.37). In a Cox-regression model, the adjusted hazard ratio (HR) of dying 5 days after GNB-LOS onset vs uninfected controls was 3.7 (CI: 1.2–11.2), but no increased hazard was seen in GNB-LOS versus suspected LOS (Table 3). The cumulative survival rate, shown in the Kaplan-Meier curves for 5 days survival, is illustrated in Fig. 2.

**Table 3:** Cox-regression survival analysis of hazard rate (HR) at 5 days after onset of LOS symptoms

	Case—uninfected*			Case—suspected*			Suspect—uninfected*		
Group	HR	95% CI	*p* Value	HR	95% CI	*p* Value	HR	95% CI	*p* Value
5 days ALL crude	5.5	2.4–12.8	< 0.001	4.5	1.9–10.7	0.001	1.2	0.3–4.9	0.76
5 days LOS crude	5.8	2.2–15.2	< 0.001	3.2	1.0–10.0	0.039	1.8	0.5–6.3	0.37
5 days LOS adjusted^#^	3.7	1.2–11.2	0.019	2.7	0.8–8.8	0.095	1.4	0.4–5.4	0.65

*Reference group

^#^The analyses are adjusted for gestational age, gender, prenatal steroids, mechanical ventilation, and necrotizing enterocolitis (NEC)

The uninfected group is matched to the same days of life when the GNB-sepsis case was diagnosed

### Morbidity

The GNB-EOS group differed in univariate analysis from the uninfected controls with a higher proportion of IVH grades 3–4 (15% vs 2%, p = 0.004) and ROP 3–4 (12% vs 1%, *p* = 0.01). No difference was seen regarding BPD. Verified GNB-LOS differed from uninfected controls regarding ROP 3–4 (14% vs 5%, *p* = 0.019), but not regarding IVH 3–4 and BPD (Table 1). Morbidity analyses with logistic regression for GNB-EOS showed an OR for the composite outcome measure death/IVH3–4 of 7.5 (CI: 1.29–43.4) compared to suspect EOS and 5.2 (CI: 1.17–23.4) compared to uninfected controls. For GNB-LOS OR was 3.0 (CI: 1.30–6.76) for death/ROP3–4 compared to suspect LOS and 6.3 (CI: 2.79–14.0) compared to uninfected controls.

For GNB-LOS, the OR for the composite variable death/BPD was 3.8 (CI: 1.68–8.67) compared to uninfected controls, but no difference was seen compared to suspect LOS.

### Bacterial characteristics and antibiotic resistance

All 107 GNB from confirmed positive blood cultures are presented in Online Resource 2. The majority belonged to the order *Enterobacterales*, comprising *E. coli*, *K. pneumoniae, Enterobacter* spp., and *S. marcescens.* Three other gram-negative species were represented: *A. baumannii*, *P. aeruginosa*, and *H. influenzae*. Proportions of deaths from GNB-EOS and GNB-LOS and the causing pathogen can be seen in Online Resource 3. Multidrug resistance was observed in 3/47*E. coli* and 2/20*K. pneumoniae* and 2/14*E. cloacae*. The antibiotic resistance pattern of all isolates is presented in Table 4. The genomic characterization of the invasive isolates that infected one-third of the neonates in the study can be seen in Online Resource 4. Of all GNB strains, 7/107 were resistant to at least two groups of antimicrobials, and all were susceptible to carbapenems.

**Table 4:** Summary of antibiogram of the 107 Gram-negative isolates from all neonates included in the study

Gram-negative bacteria	Number of isolates	Ratio of resistant isolates
*Enterobacterales*		
*E.coli*	47	2/47 GEN 3/47 TSU, CTX, CFZ 7/47 TSU
*K. pneumoniae*	20	2/20 TSU, CTX, CTZ, GEN, CIP
*K. aerogenes*	2	1/2 TSU
*K. oxytoca*	4	0
*E. cloacae*	14	1/14 GEN 2/14 CTX, CFZ
*S. marcescens*	10	0
*C. koseri*	1	0
Non-*Enterobacterales* genera		
*Acinetobacter*		
*A. baumannii*	3	0
*Pseudomonas*		
*P. aeruginosa*	4	0
*Haemophilus*		
*H. influenzae*	2	0

All isolates were susceptibility tested for the following: *GEN* gentamicin, *AMI* amikacin, *TSU* trimethoprim-sulfamethoxazole, *CTX* cefotaxime, *CFZ* ceftazidime, *CIP* ciprofloxacin, *IMI* imipenem, *MER* meropenem, *ERT* ertapenem, and *PT* piperacillin-tazobactam

## Discussion

Gram-negative sepsis is an uncommon but serious disorder in the neonate, especially in the premature born [4–6, 8, 32]. In this 11-year retrospective study, we sought to describe GNB-sepsis by reporting the incidence, subsequent mortality, and morbidity and to compare it to suspected sepsis and uninfected controls in neonates in our region.

The incidence of neonatal GNB-sepsis in the region was 0.35/1000 live born neonates and remained unchanged during the study period. The incidence of GNB-EOS was 0.11/1000 live births which is about half of what recently has been reported from the western part of Sweden, where the incidence of GNB-EOS was 0.25/1000 live births [9]. The difference is substantial but might be influenced by methodological differences. The incidence of GNB-LOS was 0.24/1000 live births and has not been previously described in a Swedish context. The incidence of 1.4 *E.coli*-LOS per 1000 NICU admissions was about half that reported in studies from other high-income countries [3, 5, 8].

We found that the need for intensive care interventions differed between the groups. The GNB-LOS group had significantly more days of supportive intensive care compared to uninfected controls, but not to suspected-LOS. These invasive measures could be risk factors for LOS but also the consequences of infection. As well neonates with suspected sepsis needed more intensive care in terms of mechanical ventilation, parenteral nutrition, and central catheters, than the uninfected controls.

The antenatal factors delivery by caesarean section and exposure to prenatal antibiotics occurred more frequently in infants with GNB-LOS than in infants with suspected sepsis or in uninfected controls. A dysbiotic neonatal intestinal microbiota due to C-sectionand/or use of antibiotics has previously been associated as a risk factor for neonatal LOS. The suggested biological rationale is that an altered first-colonizing microbiota cannot confer protection against bacterial translocation in the neonatal intestine [33, 34].

We found GNB-sepsis to be a great risk factor for mortality and show the in-hospital mortality rate to be 28% of all GNB-sepsis cases. The in-hospital mortality rate was more than 2.3 times higher among the infants with GNB-LOS compared to those with GNB-EOS, which possibly reflects the fact that the LOS group were more premature, had lower BW, had more co-morbidities and a longer duration of hospital care. Prenatal steroids have been shown to be protective against a number of morbidities in preterm infants [35], and in this study it was protective against death from GNB-LOS but not from GNB-EOS.

When adjusted for confounders, the GNB-LOS group’s in-hospital mortality was 3.9 times higher compared to uninfected controls. We found no statistical differences in in-hospital mortality between the other control groups.

Many studies on neonatal sepsis present crude mortality after a positive blood culture. However, autopsy completion is infrequently performed. We have tried to relate the sepsis episode with sepsis-related mortality and calculated the 5 days CFR. The 5 days CFR for GNB-EOS was 15% and for GNB-LOS 17%. GNB-LOS was most common (70%) in the lower GA (≤ 28 weeks), and the CFR was as high as 35% in this group.

From the survival analysis, we concluded that the adjusted hazard for dying within 5 days from the GNB-LOS onset was four times greater than if the neonate was uninfected. There was no statistical significance in the adjusted Cox-regression analysis in comparing the other groups with each other, which possibly might reflect a type II error and the small number of observations. The Kaplan-Meier curve gives us the indication that suspected-LOS is associated with a greater hazard of surviving than in uninfected, but we could not show that statistically. Causal data on reasons for death in the suspected sepsis and uninfected group were not analyzed but could be explained by the most common non-infectious causes of death in the NICU such as respiratory failure, asphyxia, IVH, metabolic disease, and lethal genetic syndromes.

Without doubt, culture proven GNB-LOS is related to an increased risk of mortality and morbidity, as previously reported [3, 6, 8, 32]. However, the power of this study is not sufficient to find out whether suspect sepsis is an entity of its own or just sepsis not possible to detect by culture. Studies conducted in high-income countries report suspected sepsis to be 6–16 times as more common than culture proven sepsis [14, 18].

We could not draw conclusions about the association between GNB-sepsis and severe complications of preterm birth such as BPD and ROP 3–4, as the most severely ill patients died before they could be validated for these conditions. IVH occurs early during the same time frame as GNB-EOS and was also overrepresented in GNB-EOS compared to neonates with suspected sepsis and the uninfected. As IVH often occurs before the onset of GNB-LOS, we did not analyze it in this context. Both ROP 3–4 and BPD was associated with GNB-LOS.

*E.coli* was the most common pathogen causing GNB-LOS with a 5 days CFR of 9%. The highest 5 days CFR (33%) was caused by the *Enterobacter* spp. We could not statistically relate specific pathogens to mortality which is an important issue for the clinician.

The rate of antibiotic resistant bacteria in our study was low compared to studies from other settings [36, 37]. In a recent retrospective study between 2009 and 2017 from the USA, a mean of 5% ESBL-producing*E.coli* was seen in a large cohort (*n* = 733) of neonatal *E-coli* sepsis [38]. The proportion of all ESBL-producing*Enterobacterales* in our study, with a smaller sample size, was 7/107 (6.5%) and is still considered low. The low incidence of GNB-LOS and AMR could be the result of long-standing efforts in infection control and antimicrobial stewardship [39, 40]. In 2012, we changed our empiric aminoglycoside from gentamicin or netilmicin to amikacin due to repeatedly small outbreaks of gentamicin-resistant*E.coli* in the region. After that, we could not see any high rates resistance to amikacin or third generation cephalosporins that would lead to any change in the empiric therapy.

One strength of the study is its population-based approach as it covers almost all 310,091 infants born in the Stockholm region during the study period. Another strength of the study is that all medical records from the patients with GNB-sepsis, suspected sepsis, and controls were validated against medical records. All data was validated against medical records because between 2006 and 2010, there were no predefined sepsis criteria and data completeness in SNQ regarding causative agents was low in the study region. Sepsis criteria have in later years been standardized, and reporting to SNQ has been changed from retrospective summaries to web-based uploads on a daily basis. In later years, SNQ has been shown to exhibit similar or higher completeness for neonatal sepsis as the Swedish Medical Birth register which is considered to be very high [41] .

The limitations of the study are related to the retrospective design and, despite covering all cases during an 11-year period in an area with more than 2 million inhabitants, the small sample size. The procedure of matching the controls to each sepsis case has been done as accurately as possible. The physiological vulnerability of the neonate in different gestational ages is the most important variable for matching. The size of the cohort makes it impossible to match for more morbidities and is therefore a limitation of the study.

## Conclusion

We conclude that GNB-sepsis is rare but it remains a serious threat to neonatal patients in the region. GNB-sepsis is a risk factor for neonatal mortality compared to suspect sepsis and uninfected controls. We found a lower incidence of GNB-EOS than previously described in Sweden and other high-income settings, and for the first time, we present the incidence of GNB-LOS in Sweden. The GNB-EOS or GNB-LOS incidence did not change during the study period. The incidence of AMR was low, the AMR pattern did not reveal any highly resistant strains, and the incidence did not change over time. This is reassuring as the current empiric therapy against bacterial sepsis of unknown origin appears to be relevant despite its use over a long period of time.

## Supplementary Information

ESM 1 (DOCX 51.1 kb)

ESM 2 (DOCX 258 kb)

ESM 3 (DOCX 51.3 kb)

ESM 4 (DOCX 27.1 kb)

## Data Availability

The dataset is available on your request.
