# Supplementary material for: A decade of neonatal sepsis caused by gram-negative bacilli—a retrospective matched cohort study
Source: Eur J Clin Microbiol Infect Dis. 2021 Mar 24;40(9):1803–13. doi: 10.1007/s10096-021-04211-8 (PMC8346411; doi:10.1007/s10096-021-04211-8)
Supplement: Supplementary file 1 — (DOCX 51.1 kb) [file 10096_2021_4211_MOESM1_ESM.docx]

**Online Resource 1.** Case fatality rates of GNB-EOS and GNB-LOS in different gestational ages.

|  | No  GNB-sepsis | No of  GNB-EOS | EOS death within  5 days | EOS  death within  NICU | No of GNB-LOS | LOS death within  5 days | LOS death within  NICU |
| --- | --- | --- | --- | --- | --- | --- | --- |
| **Gestational age (weeks)** |  |  |  |  |  |  |  |
| *22-24* | 21 | 5 | 2 (40%) | 2 (40%) | 16 | 6/16 (37.5%) | 8/16 (50%) |
| *25-28* | 40 | 4 | 0 | 0 | 36 | 5/36 (13.8%) | 10/36 (28%) |
| *29-32* | 24 | 7 | 1 (14.2%) | 1 (14.2%) | 17 | 1/17 (5.8%) | 6/17 (35%) |
| *33-36* | 8 | 5 | 1 (20%) | 1 (20%) | 3 | 1/3 (33%) | 1/3 (33%) |
| *≥37* | 14 | 12 | 1 (8.3%) | 1 (8.3%) | 2 | 0 | 0 |
| **All GNB cases** | 107 | 33 | 5 (15%) | 5 (15%) | 74 | 13/74 (17.5)% | 25/74 (34%) |
