# Supplementary material for: A decade of neonatal sepsis caused by gram-negative bacilli—a retrospective matched cohort study
Source: Eur J Clin Microbiol Infect Dis. 2021 Mar 24;40(9):1803–13. doi: 10.1007/s10096-021-04211-8 (PMC8346411; doi:10.1007/s10096-021-04211-8)
Supplement: Supplementary file 2 — (DOCX 258 kb) [file 10096_2021_4211_MOESM2_ESM.docx]

**Online resource 2**: A total of 804 episodes of culture confirmed neonatal sepsis episodes. All confirmed
Gram-negative pathogens and the case fatality per pathogen is presented.
^a^ Due to missing data from four neonates and two apathogen Neisseria, 107 of 113 confirmed Gram-negative pathogens are presented here.
