# Supplementary material for: A decade of neonatal sepsis caused by gram-negative bacilli—a retrospective matched cohort study
Source: Eur J Clin Microbiol Infect Dis. 2021 Mar 24;40(9):1803–13. doi: 10.1007/s10096-021-04211-8 (PMC8346411; doi:10.1007/s10096-021-04211-8)
Supplement: Supplementary file 3 — (DOCX 51.3 kb) [file 10096_2021_4211_MOESM3_ESM.docx]

**Online Resource 3.** Case fatality rate of pathogen specific EOS and LOS in 107 neonates.

| **Confirmed Gram-negative pathogens** | Total no of GNB-sepsis | No of  GNB-EOS | EOS  case fatality rate 5 days  (%) | EOS  case fatality rate before discharge  (%) | No of GNB-LOS | LOS case fatality rate  5 days  (%) | LOS case fatality rate  NICU  (%) |
| --- | --- | --- | --- | --- | --- | --- | --- |
| *Escherichia coli* | **47** | **24** | 2/24 (8.3) | 3/24 (12.5) | **23** | 2/23 (9) | 3/23(13) |
| *Klebsiella* spp | **24** | **5** | 1/5 (20) | 1/5 (20) | **19** | 3/19 (16) | 4/19 (21) |
| *Enterobacter* spp | **16** | **1** | 0 | 0 | **15** | 5/15 (33) | 6/15 (40) |
| *Serratia marcescens* | **10** | **0** | - | - | **10** | 2/10 (20) | 4/10 (40) |
| *Acinetobacter baumannii* | **3** | **0** | - | - | **3** | 0 | 0 |
| *Pseudomonas aeruginosa* | **4** | **1** | 1/1 (100) | 1/1 (100) | **3** | 0 | 1/3 (33) |
| *Haemophilus influenzae* | **2** | **2** | 0 | 0 | **0** | - | - |
| *Citrobacter koseri* | **1** | **0** | - | - | **1** | 1/1 (100) | 1/1 (100) |
