# Supplementary material for: A decade of neonatal sepsis caused by gram-negative bacilli—a retrospective matched cohort study
Source: Eur J Clin Microbiol Infect Dis. 2021 Mar 24;40(9):1803–13. doi: 10.1007/s10096-021-04211-8 (PMC8346411; doi:10.1007/s10096-021-04211-8)
Supplement: Supplementary file 4 — (DOCX 27.1 kb) [file 10096_2021_4211_MOESM4_ESM.docx]

**Online resource 4.** Results from WGS, MLST and SNP-analysis on 33 isolates causing neonatal GNB-sepsis in Stockholm during the years 2010 and 2013-2016.

The MLST data showed a high diversity of sequence types (STs) in *E. coli* (n=12*)*, *E. cloacae* (n=4) and *K. pneumoniae* (n=4)*.* The two *K. oxytoca* strains had the same sequence type, ST176, but differed with 276 SNPs and were thus unrelated. In the *E. coli* group, three patients had EC ST95 and two patients EC ST357, but the SNP-analysis revealed no close genetic relation within these two STs. *K. pneumoniae* had no related STs. No MLST scheme was available for *S. marcescens*, but with SNP-typing a high level of genetic similarity in three patients could be detected, all hospitalized in the same NICU between February and May 2016.
The *S. marcescens* strain differed in 1-3 SNPs.

| **Bacterial species** | **GA** (w+d) | **NICU site** (month/year) | **MLST** | **SNP- difference*** | | **Resistance genes** | **Phenotypic resistance°** |
| --- | --- | --- | --- | --- | --- | --- | --- |
| *E. coli* | 26+0 | K-S 0116 | ST95 | 1876-5626 | *mdf(A)* | | S to all Ab |
| *E. coli* | 38+0 | SÖS 0814 | ST95 | 1876-5626 | *bla*_TEM-1C_, *mdf(A)* | | S to all Ab |
| *E. coli* | 41+2 | SÖS 1013 | ST95 | 1876-5626 | *mdf(A)* | | S to all Ab |
| *E. coli* | 23+2 | K-S 1015 | ST10 |  | *aadA1, aph(3")-Ib, aph(6)-Id,* *bla* _TEM-1B_, *mdf(A), catA1, sul1, sul2, tet(A), dfrA1* | | S to all Ab |
| *E. coli* | 42+1 | K-S 0413 | ST12 |  | *aph((3´)-Ia, aph(6)-Id, mdf(A), sul2, tet(A)* | | S to all Ab |
| *E. coli* | 29+4 | K-HS 0116 | ST96 |  | *aac(3)-Iid,* *bla*_TEM-1B_, *mdf(A)* | | R to GEN |
| *E. coli* | 26+6 | K-S 1014 | ST117 |  | *aph(3")-Ib,* *bla*_TEM-1C_, *mdf(A), sul2, tet(A)* | | S to all Ab |
| *E. coli* | 30+4 | K-S 0114 | ST120 |  | *aph(6)-Id,* *bla*_TEM-1B_, *mdf(A), catA1,* *tet(B), dfrA17* | | R to TSU |
| *E. coli* | 24+2 | K-S 0415 | ST297 |  | *aph(6)-Id,* *bla*_TEM-1B_, *mdf(A), catA1,* *tet(B), dfrA17* | | R to TSU |
| *E. coli* | 30+0 | K-S 0715 | ST1196 |  | *aadA1,* *bla*_CTX-M-27_, *bla*_OXA-10_, *mdf(A),* *mph(A), cmlA1, floR, ARR-2, sul2, tet(A), dfrA14* | | R to AMC, CEX, TRI, TSU, CTX, CFZ, CIP |
| *E. coli* | 39+2 | SÖS 0513 | ST2144 |  | *mdf(A)* | | S to all Ab |
| *E. coli* | 32+0 | SÖS 0415 | ST3857 |  | *aadA1,* *bla*_CTX-M-15_, *bla*_CTX-M-218_, *mdf(A),* *qnrS1, dfrA1* | | R to CEX, TRI, CTX, CFZ |
| *E. coli* | 38+6† | K-HS 0716 | ST8186 |  | *mdf(A)* | | S to all Ab |
| *E. coli* | 30+0 | SÖS 1216 | ST357 | >204 | *mdf(A)* | | S to all Ab |
| *E. coli* | 40+2 | K-HS 0513 | ST357 | >204 | *mdf(A)* | | S to all Ab |
| *E. clo* | 24+0 | K-S 1210 | ST50 |  | *bla*_ACT-15_ | | S to all Ab |
| *E. clo* | 36+5† | K-HS 0616 | ST662 |  | *bla*_ACT-7_ | | S to all Ab |
| *E. clo* | 26+6 | K-DS 0710 | ST4858 |  | *bla*_OKP_, *aph* | | S to all Ab |
| *E. clo* | 23+6† | K-S 0310 | Novel | 7 | *bla*_ACT-1_, *bla*_ACT-2_ | | S to all Ab |
| *E. clo* | 23+2† | K-S 0310 | Novel | 7 | *bla*_ACT-1_, *bla*_ACT-2_ | | S to all Ab |
| *K. oxy* | 27+3 | SÖS 0313 | ST176 | 274 | *bla*_OXY-2-5_ | | S to all Ab |
| *K. oxy* | 26+2 | K-S 1210 | ST176 | 274 | *bla*_OXY-2-5_ | | S to all Ab |
| *K. pne* | 29+2 | SÖS 0715 | ST133 |  | *bla*_SHV-75_, *fosA, oqxA, oqxB* | | S to all Ab |
| *K. pne* | 28+5† | K-S 0310 | ST299 |  | *bla*_SHV-199_, *fosA* | | S to all Ab |
| *K. pne* | 35+2 | K-DS 1114 | ST2703 |  | *bla*_SHV-199_, *fosF, oqxA, oqxB* | | S to all Ab |
| *K. pne* | 26+1† | K-S 0210 | ST4059 |  | *bla*_LEN16_ | | S to all Ab |
| *S. mar* | 32+2† | K-S 0716 | NA | >121938 | *aac(6')-Ic,* *bla*_SRT-1_,*srm(B), oqxB, tet(41)* | | S to all Ab |
| *S. mar* | 29+6† | K-S 0216 | NA | 0-1 | *aac(6')-Ic,* *bla*_SRT-1_ | | S to all Ab |
| *S. mar* | 25+3 | K-S 0216 | NA | 0-1 | *aac(6')-Ic,* *bla*_SRT-1_ | | S to all Ab |
| *S. mar* | 25+3 | K-S 0216 | NA | 0-1 | *aac(6')-Ic,* *bla*_SRT-1_ | | S to all Ab |
| *S. mar* | 24+2 | K-HS 0815 | NA | >27911 | *aac(6')-Ic,* *bla*_SRT-1_ | | S to all Ab |
| *S. mar* | 26+5 | SÖS 0514 | NA | >20423 | *aac(6')-Ic,* *bla*_SRT-1_ | | S to all Ab |
| *C. kos* | 32+5† | SÖS 0616 | NA |  | *bla*_MAL-1_ | | R to CEX |

WGS- whole-genome sequencing, MLST- multilocus sequence typing. SNP-Single-nucleotide polymorphisms. NICU-site; K-S is Karolinska Solna, K-HS is Karolinska Huddinge, K-DS is Karolinska Danderyd, SÖS is Södersjukhuset. GA-Gestational age (weeks and age).
Grey shadow- same sequence type and further analyzed with SNP-analysis.
*Number of base pairs that differ between a reference isolate with the same ST.
If <4 SNP difference, the isolate is considered to be the same strain. †Death during NICU-stay.
°All Enterobacterales was tested for cefotaxime, ceftazidime, piperacillin-tazobactam, imipenem, meropenem, ertapenem, ciprofloxacin, trimethoprim-sulfametoxazol, gentamicin, amikacin.
